# COMPARE FRAILTY IN OLDER ADULTS AND NONOLDER ADULTS WITH HEART FAILURE

**DOI:** 10.1093/geroni/igae098.3295

**Published:** 2024-12-31

**Authors:** Jenny Hsieh, Pi-Ling Chou

**Affiliations:** University of Washington, Seattle, Washington, United States; Kaohsiung Medical University, Kaohsiung, Kaohsiung, Taiwan (Republic of China)

## Abstract

Frailty is commonly presented among patients with chronic diseases, including heart failure(HF), leading to poor prognosis. However, the association between age and frailty in patients with HF remains unclear. Therefore, our study aims to compare the difference in frailty status in older and non-older adults with chronic HF symptoms. A cross-sectional study was conducted, where data was collected between January 1, 2023, and January 31, 2024. Participants were recruited from cardiology departments of two hospitals in southern Taiwan (N=110). Instruments measuring frailty included the Frailty Phenotype Criteria (FPC) and Clinical Frailty Scale(CFS). Data analysis was conducted using R version 4.3.2. In our study, we observed pre-frailty and frailty in older adults with proportions of 49.1% (n=26) and 22.6% (n=12) according to FPC, respectively. For non-older adults, the proportions were 36.4% (n=16) and 18.1% (n=5), respectively. The CFS revealed that 50.8% (n=31) of older adults exhibited frailty, while 38.8% (n=19) of non-older adults did. No significant difference was observed in frailty status between older and non-older adults, as indicated by both the FPC (p = 0.21) and the CFS (p = 0.29). The findings suggested a high prevalence of frailty in patients with heart failure, irrespective of age group. Our results indicated that frailty was a functional decline not limited to older adults. This study highlighted the importance of frailty assessments in patients with heart failure symptoms across all age groups, providing an evidence base to innovate potential strategies to promote improvement in prognosis.

